# Analysis of the Scenarios of Use of an Innovative Technology for the Fast and Nondestructive Characterization of Viscoelastic Materials in the Tires Field

**DOI:** 10.3390/s24041136

**Published:** 2024-02-09

**Authors:** Flavio Farroni, Francesco Timpone, Andrea Genovese

**Affiliations:** 1 Department of Industrial Engineering, University of Naples Federico II, 80125 Naples, Italy; flavio.farroni@unina.it (F.F.); francesco.timpone@unina.it (F.T.); 2 VESevo Smart Technologies SRL, 80125 Naples, Italy

**Keywords:** measurement system, viscoelastic materials, rubber characterization, tire analysis, nondestructive testing, quality control, polymers science, real-time monitoring

## Abstract

The properties of tires related to their viscoelastic behavior have a significant impact in the field of vehicle dynamics. They affect the performance and safety of a vehicle based on how they change when the tire performs in variable thermal conditions, interacts with various kinds of road surfaces, and accumulates mileage over time. To analyze and understand such properties of viscoelastic materials, destructive tests like dynamic mechanical analysis (DMA) are used, which make the tire unusable after the test; these are usually carried out on specimens cut from the zone of interest. The development of an innovative testing methodology connected to a hardware device called VESevo allows the characterization of the viscoelastic properties of tire compounds belonging to tread or other parts in a fast and nondestructive way. This new device provides valuable information about the evolution of the tire’s viscoelastic properties, allowing it to monitor them throughout the whole lifecycle. In the paper, an overview of the possible sensitivities that can be investigated thanks to the VESevo is provided: The tread viscoelasticity was characterized and monitored for several tire tread compounds, over tire mileage, over tread thermal curing cycles, and as an index of the tread quality and uniformity in production. Preliminary results were collected and are presented. In the final paragraph, further recent applications developed from the tire field, which are not directly related, are reported.

## 1. Introduction

The performance and safety of tires are largely dependent on the viscoelastic properties of the tire tread. These properties determine the tire’s ability to absorb shocks, deform under load, and recover its shape when unloaded [1,2]. As such, the viscoelastic properties of tire tread are critical to ensure optimal friction performance [3,4,5], which is crucial for road safety, especially in adverse weather conditions [6]. Conventionally, the viscoelastic properties of tire tread are determined using destructive mechanical testing methods, such as dynamic mechanical analysis (DMA) [7] or stress relaxation tests [8]. However, these methods are time-consuming, expensive, and cause unavoidable damage to the tire tread, rendering the tire unusable. Moreover, the results obtained from these methods are often influenced by sample preparation (because the cut specimens often modify the internal stress/strain balance), the testing conditions (because the specimens clamping cannot be optimal, or the DMA controls since they are not precise enough because the material changes its own properties during the test), and by geometry (because the specimens have to be cut in very precise shapes, which is quite complex for such deformable bodies), leading to large variations in the measured parameters [9].

To overcome these challenges, nondestructive and innovative methods for measuring the viscoelastic properties of tire tread have been developed: among these methods, spectroscopy [10] and ultrasounds [11] are the most used. However, each of them is not yet consolidated enough as a DMA, owing to high costs and the necessity of bulky and not portable measurement layouts.

In this paper, the results from the application of a novel methodology based on a simple mechanical principle [12,13] linked to the analysis of the indentation caused by a rod-shape micro punch, falling under the mere effect of gravity from a specific distance from the tested material, are presented. The device providing such measurements, and, consequently, the full “master curve” material characterization in terms of “storage modulus” and “loss factor” [14], has been named VESevo [15] (acronym for Viscoelasticity Evaluation System EVO) and has been preliminary developed to satisfy the requests of motorsport players, requiring a portable system able to provide robust viscoelastic data from the tire being used for races, and that for rules constraints cannot be analyzed using the common destructive techniques. Once adopted by several racing teams in many international categories, the device has been progressively discovered by tire manufacturers and, in general, by plastic materials producers for its capability to provide a nondestructive and precise measurement of the viscoelastic properties of the tested materials, becoming particularly useful in real-time quality control assessment on the production lines.

In the last couple of years, the VESevo technology has been used in a wide range of applications, each usually proposed by new kinds of users, proposing procedures linked to their areas of interest:

- Providing measurements to objectivize the differences among the various compounds used for tire treads (with important impacts on determining the optimal thermal window in which each compound has to be used), getting data for FEA (finite element analysis) [16], CFD (computational fluid dynamics) [17], friction [18], and wear [19] models, both in motorsport and passenger contexts;
- assessing the optimal vulcanization and manufacturing setup conditions, with a direct evaluation of their effects, from the tire just produced to its progressive degradation in the aging process acting in the lifecycle, or helping to define the optimal “curing cycles”; the motorsport teams are used to apply to the tires to enhance their adhesive and hysteretic frictional attitudes;
- monitoring the evolution of the viscoelastic characteristics due to wear (conceived as both progressive tread thickness reduction [20] under tangential interaction stresses and progressive mechanical degradation due to hysteretic cycles and chemical decay [21]) during the whole life of the tested tire, allowing to correlate the energy provided to it (for frictional forces, for rolling resistance cycles, for UV radiation effect) with the direct effects on the material, observable from its first instant of life, to the ultimate phase of use;
- improving the effectiveness of the on-site quality control methodologies [22], acting as a sort of “fast and portable DMA” directly on the tire production line, checking the viscoelastic uniformity along the circumferential and transversal direction for car, truck, aircraft, motorcycle, and bike tires, and for slick or ribbed tread patterns.

All the described scenarios of use allowed the collection of data, which will be analyzed in the paper, connecting the highlighted behaviors with the physical phenomena causing them. The implications of this study are significant for the tire and transportation industries and for global road safety improvement towards the progressive implementation of the “Vision Zero” strategy [23] and the development of enhanced tire-centered real-time onboard control algorithms [24]. The viscoelastic properties of tire tread obtained through this methodology can be utilized to develop new tire formulations with improved friction, durability, and safety performance. Moreover, the method can be utilized for quality control monitoring of tire production, ensuring consistent and uniform tire performances, and minimizing unexpected failures and wastes in the manufacturing process, with improvements in the sustainability cycle. Further fields of application are currently under investigation, and the results will be the object of further papers on the same research topic.

## 2. Basic Concepts on Viscoelasticity and Friction Principles for Rubber Materials

A viscoelastic material is a deformable material with a behavior that lies between a viscous liquid and an elastic solid. The feature that differentiates these materials from others is that, while in the case of elastic solids and viscous fluids, the answer to excitation or instantaneous deformation is instantaneous and independent of time, in the case of viscoelastic materials, it is a function of time [25]. This means that the stress–strain relationship is defined by a complex dynamic modulus, described as the overall amount of resistance to the deformation of the compound:

(1) $$\frac{\sigma (t)}{\varepsilon (t)}=E^{*}=E_{1}+{iE}_{2}$$

(2) $$tan\left.\;\delta \right.=\frac{E_{2}}{E_{1}}$$

The complex dynamic modulus has a real and an imaginary part: The first is defined as the “storage modulus” (E′) and is related to the elasticity of the material, linked to its ability to store energy; the second is called “loss modulus” (E″) and is associated with the capability of the material to dissipate energy, usually as heat. The ratio between the loss modulus and the storage modulus results in an index named loss factor (tan δ), which indicates the material’s overall damping potential, which is one of the key parameters in the study of tire friction.

**Figure 1:**
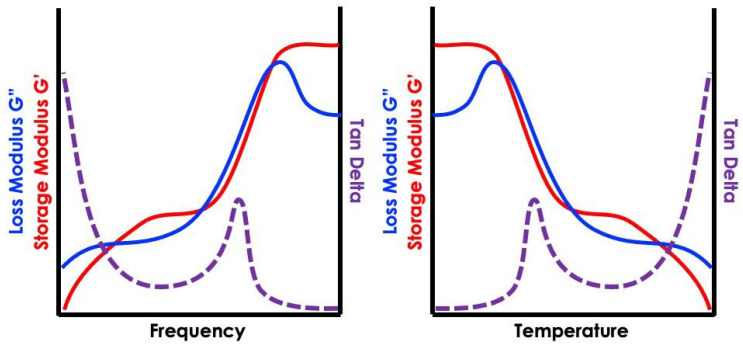
Storage and loss modulus qualitative trend with respect to temperature and frequency.

The behavior of viscoelastic materials when a stress/strain is applied to them is strongly dependent on both the excitation frequency and temperature, as reported in Figure 1, which qualitatively represents the so-called “master curves” behavior under the application of thermal and frequency variations. Such two physical quantities can be mutually correlated by applying an equation defined by the authors William, Landel, and Ferry [26,27], indeed, known as W.L.F. law, which reproduces the so-called temperature/frequency rubber materials superposition principle.

**Figure 2:**
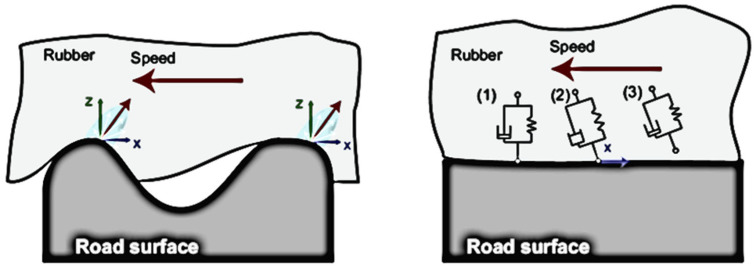
Friction phenomena: road hysteretic friction effect due to indentation on the left; molecular adhesion on the right [29].

Knowing the viscoelastic properties is a fundamental advantage in defining strategies and methods to maximize road grip, whose definition is “the maximum available coefficient of friction between the surface of the tire and the surface of the road” (or of any other counter-surface in contact with the tread). Such friction depends on several factors [28], such as the roughness of the road/track, the relative speed and the contact pressure between the tire and road, the tire temperature and wear level, and, of course, the viscoelastic characteristics of the tread. As concerns the factors mainly taking part in friction as considered in the vehicle dynamics field, two mechanisms, shown in Figure 2, are involved at the rubber–road interface [29]:

- The first is the frequency excitation due to the road texture. The rubber is deformed when it slips over the rough unevenness of the road, whose size varies from centimeters (macrotexture) to microns (microtexture) [30], dissipating an amount of power different on each frequency range. This mechanism, usually described as hysteretic friction, is linked to the penetration of road roughness indenters into the rubber of the tire tread and is responsible for the solicitations shifting at various frequencies of the polymers’ “master curves” following the W.L.F. law.
- The second is due to molecular adhesion, which comes into play at smaller dimensional scales and is deeply linked to the chemical attitudes of the material in contact that creates Van der Vaals bonds.

In both mechanisms, the viscoelastic properties of the rubber, particularly its attitude to dissipate energy (linked to the loss factor) in the hysteretic grip [31] and its attitude to “stick” to the counter-surface (linked to the storage modulus) in the adhesive grip [32], play an important role in affecting the overall tire tread performance.

## 3. VESevo Nondestructive Testing Procedure

**Figure 3:**
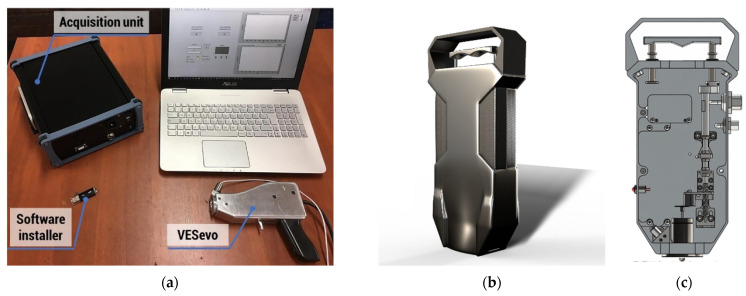
VESevo device and acquisition unit: (**a**) the prototypal version; (**b**) the latest model, with embedded acquisition unit; (**c**) the section view with internal mechanisms detailed.

The measurement system acquiring the data reported in the present work is composed of a hardware device designed to generate the proper contact between a metal indenter and the rubber material and to measure its relative motion, using software for raw data acquisition and its subsequent processing. In its preliminary version, as in Figure 3a, the acquisition system was located outside the measurement system, whereas in the latest versions, everything is embedded in the VESevo device.

The working principle of the device is based on measuring the displacement of a steel rod with a semi-spherical titanium indenter, which is free to fall and bounce on the surface of the compound being tested. A high-precision mechanical system located inside the device, as highlighted in Figure 3c, guarantees that the free drop motion of the rod always starts from the same initial position. The motion of the rod is acquired in each test by a high-frequency integrated optical sensor, and the temperature of the tested compound is measured by a compact IR pyrometer.

A standardized testing procedure has been established for conducting robust acquisitions using the VESevo. This involves (1) positioning the device vertically, either on the tire tread area for testing or on the slab; (2) raising the indenter by pulling a sliding manual trigger until a mechanical lock is engaged, ensuring a consistent starting position and velocity for each acquisition; (3) upon reaching the release height, the rod is released using the automatic magnetic system. The acquisition software then measures and displays both the rod displacement curve and the compound temperature.

**Figure 4:**
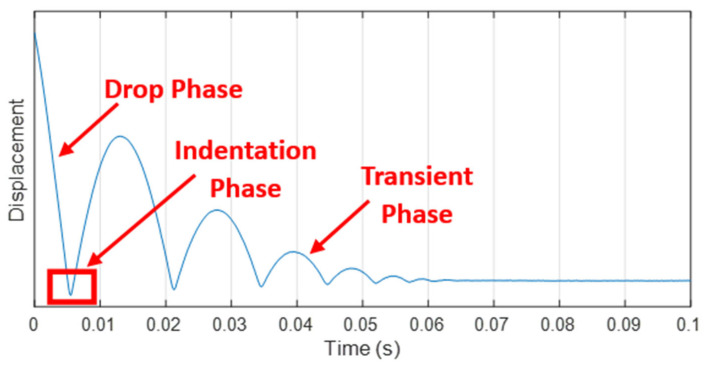
The acquired raw signal on the tire tread surface.

In Figure 4, a typical displacement signal is depicted, revealing distinct phases in the rod’s motion. These phases include the descent from the initial release height, the first indentation into the material caused by the rod penetrating the viscoelastic layer, and the subsequent transient stage during which the rod undergoes a damped oscillation before establishing a static final contact between the indenter and the tread surface, whose displacement has been found as deeply correlated to the inverse of the storage modulus E′, actually behaving as a sort of static hardness of the material.

**Figure 5:**
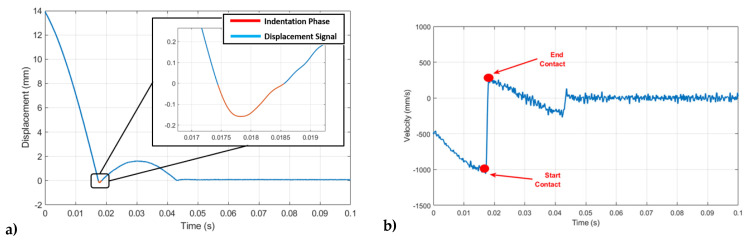
Analysis of the signals acquired by VESevo: (**a**) first indentation phase highlighted in red, in the displacement signal; (**b**) speed signal obtained as a derivative of the displacement, with highlights of the areas representing the beginning and the end of the first indentation phase.

The identification of the first indentation phase (Figure 5a), fundamental for the evaluation of the viscoelastic behavior of the material, is performed by deriving the displacement signal. The rod speed signal, obtained in such a way, is characterized by a recurring pattern, as highlighted in Figure 5b, which allows us to clearly recognize the instant representation of the start and the end of the penetration of the rod inside the rubber layer. Once such phase is identified, the variation in the squared speed before (V_s_) and after (V_f_) the indentation is proportional to the kinetic energy E_k_ dissipated within the material, as described by the following equation:

(3) $$\Delta E_{k}=K({V_{s}}^{2}-{V_{f}}^{2})$$

which is found to be proportional to the loss factor.

**Figure 6:**
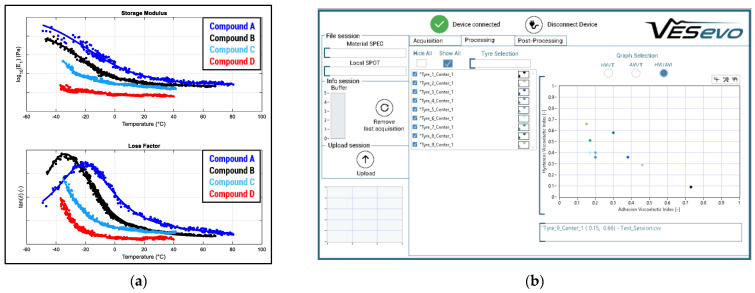
Results provided by the VESevo device and methodology: (**a**) viscoelastic master curves at variable temperatures and the 1 Hz reference frequency; (**b**) viscoelastic performance indexes useful for direct comparisons among tests carried out at fixed temperatures.

The tests, and, consequently, the viscoelastic material characterization, can be performed in two ways (Figure 6):

(A) In order to investigate the tread compounds’ behavior, obtaining the so-called material master curves (Figure 6a), dependent on the temperature, the tests were carried out by varying the sample temperature, warming or cooling the material following several methodologies (temperature-controlled chambers, thermal guns, tire thermal blankets, freezing systems, etc.).
(B) In order to perform fast evaluations on the viscoelastic performances of the tested material, acquisitions can be carried out at fixed (ambient or pre-defined reference) temperatures, providing viscoelastic indexes evaluations (Figure 6b) and comparisons among the performances showed in various areas of the same tested good/specimen or among various goods/specimens (fundamental in quality control procedures).

**Figure 7:**
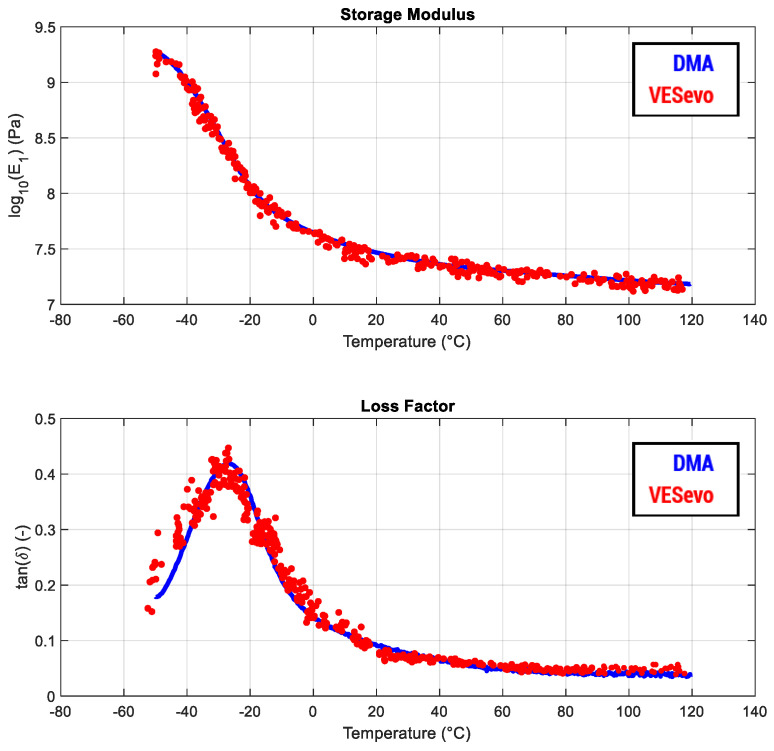
Comparison between the viscoelastic curves obtained by VESevo (in red) and DMA destructive analysis (in blue) under the same testing conditions for a reference compound.

In both cases, the data are processed using an algorithm able to analyze each acquired displacement curve at the corresponding temperature and to calculate the storage modulus and loss factor variables as the function of the quantities described in Equations (4) and (5), measured during the acquisition phase or calculated using them:

(4) $$E^{\prime }\;=f(A_{c}\;,T,K_{c})$$

(5) $$tan(\delta )=f(A_{c}\;,\omega ,\;T,S_{c})$$

in which *A_c_* is the effective contact area between the semi-spherical indenter and the compound, ω is the solicitation frequency linked to each VESevo test (due to temperature and the intrinsic response of the material), *T* is the compound temperature measured by the infrared sensor, and *K_c_* and *S_c_* are the equivalent contact stiffness and damping coefficient, respectively, which can be considered as the ones from the tested material, acting as the VESevo spring stiffness and rod guide negligible. The obtained E′ and tan δ are referred to as a 1 Hz equivalent master curve and have been validated thanks to DMA tests carried out at the same temperature and frequency conditions using the same polymer compounds (Figure 7).

## 4. Results and Related Application Fields

The described device and the related measurement procedure allow us to evaluate, as described, the viscoelastic characteristics of the viscoelastic materials in a completely nondestructive way, providing a fast and on-site methodology to assess both the behavior of a certain plastic/rubber good as concerns temperature/frequency excitement and the consistency of its production in terms of uniformity and repeatability. It opens a wide range of scenarios enabled by the possibility of following the evolution of mechanical properties during the whole lifecycle and under various conditions of use.

Moving into specific evaluations allowed by the presented methodology, various examples of the tests and the analysis that can be carried out thanks to the VESevo technology are presented in the following sections.

### 4.1. Objectivizing Tire Compounds in Motorsport Applications

**Figure 8:**
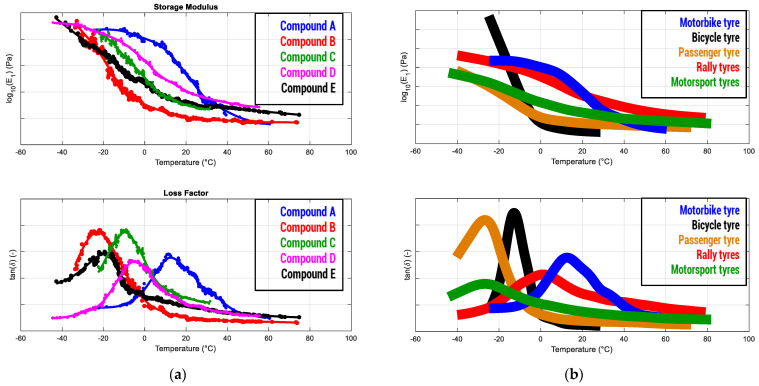
Viscoelastic master curves related to several tire compounds: (**a**) from the same motorsport category, whose “objectivation” overcomes the lack of information available when tires are identified just as having different compound “names”; (**b**) from various market segments usefully to observe the main differences among different targets of use.

Racing context has usually faced limitations due to regulations, particularly concerning tires. They are often provided by a tire manufacturing company and are used to inhibit any kind of destructive analysis on tires and, consequently, any kind of DMA or similar test. For such reason, the nondestructive analysis provided by VESevo was originally conceived to satisfy requests from motorsport. Getting objective data on the master curves related to the various tire tread compounds adopted in each racing category (Figure 8) allows us to provide a quantitative meaning, in terms of glassy transition temperature Tg and viscoelastic response, to the tread compounds names (usually just defined as “soft”, “medium”, “hard”, etc.). It is then fundamental to define the parameters feeding physical interaction, friction, and wear models, requiring clear and quantitative information on the response of the material.

**Figure 9:**
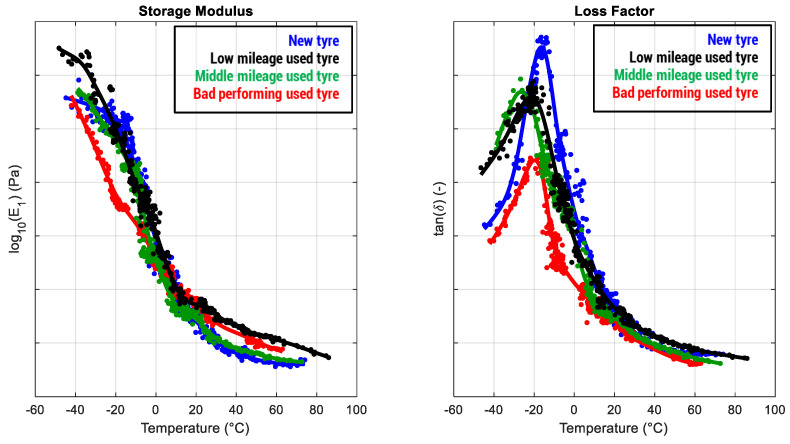
Comparison among tires exhibiting the expected performances in a driver’s subjective feedback at various mileage levels and a tire declared as “underperforming” in terms of adherence after the run (the vehicle equipping the tires had no data acquisition, and, consequently, the objective vehicle data required to validate the feedback are missing).

Moreover, objective data allow us to validate the uniformity of the tires provided by the tire manufacturer and to perform their assessment directly “in the paddock”. Finally, a methodology able to evaluate quantitatively the rubber performances in terms of exhibited adherence can represent a link between drivers’ subjectivity and actual tire characteristics, improving the correlation between vehicle telemetry data and driver’s feelings, with particular reference to progressive degradation (discussed in the following paragraph) and to tires “complained” as badly performing (as illustrated in Figure 9, where a tire defined by the driver as underperforming is compared with another exhibiting the expected behavior).

### 4.2. Evaluating Polymers’ Degradation along Lifecycle

**Figure 10:**
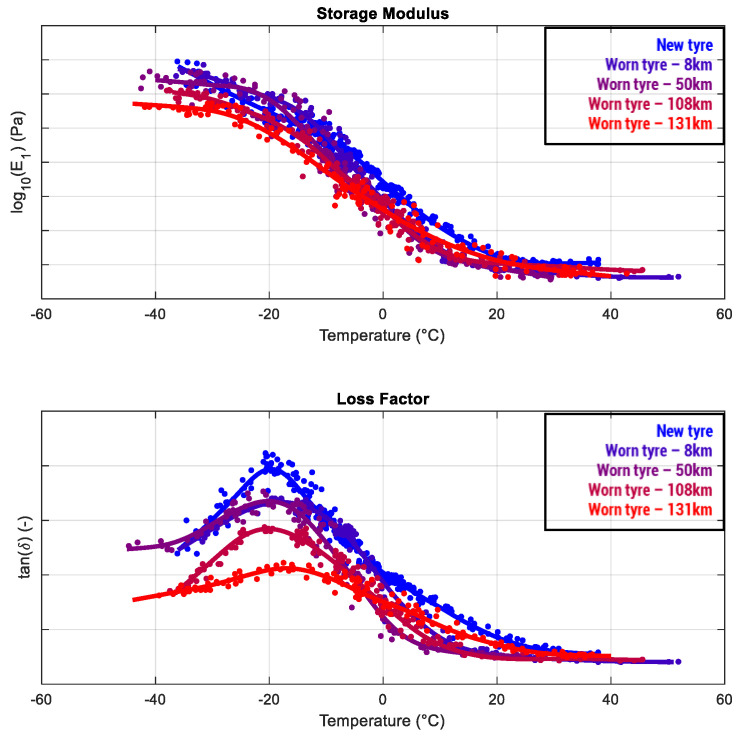
Results obtained after testing the same tire with a higher mileage level allowed for observing the progressive variations in storage modulus and loss factor.

One of the most significant impacts of the presented nondestructive testing methodology is related to the possibility of testing a certain tire (or any other plastic/rubber good) during its whole lifecycle, monitoring the effects due to its progressive use. Such a possibility is completely unavailable in common measurement methodologies due to the fact that a viscoelastic analysis would imply the removal of a “piece” of the tested object to be analyzed as a specimen by DMA or by other common destructive methods, making the whole object unusable. Moreover, a test carried out at different stages of use on several objects defined as “nominally identical” at the production phase would involve uncertainties due to the low degree of similarity among the tested objects (barely guaranteed due to the many sources of inhomogeneity during the rubber/plastic production cycle). All these limitations can be overcome following the evolution of each individual product in its whole life (Figure 10), eventually correlating the forces/energies acting on the component with the measured viscoelastic effects. An interesting behavior, often exhibited by viscoelastic materials—also highlighted in Figure 10—relates to the progressive reduction in the storage modulus, probably due to the progressive tread thickness decrease causing a lower structural stiffness, and the contemporary decrease in the loss modulus due to the reduced capability of the material to dissipate power in a thinner control volume [33]. As shown in the plots, such trends are not the same at each thermal level, and they are not always exhibited by any kind of rubber material; however, the cited phenomena can be considered as the main guidelines to explain the major effects observed at the increasing wear.

**Figure 11:**
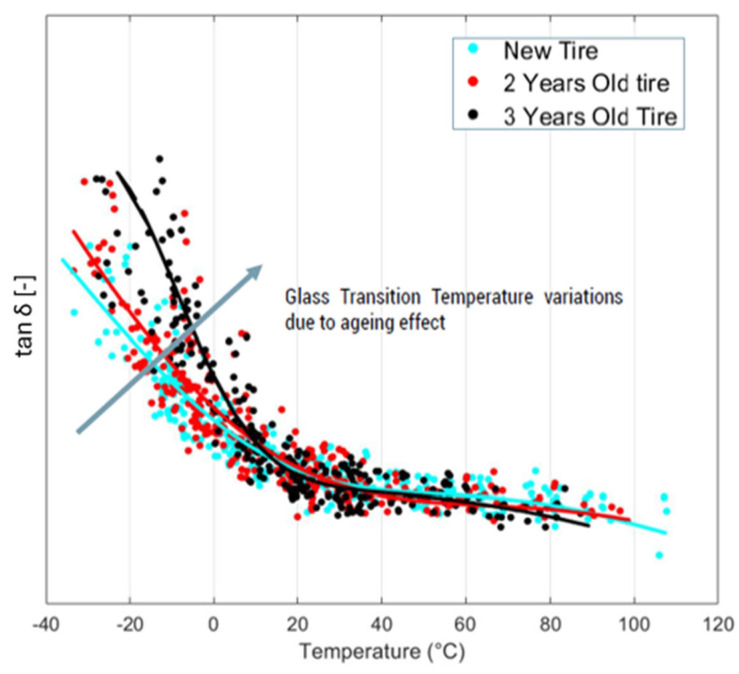
Results were obtained by testing the same tire at increasing aging levels without it being used.

Further, a specific case of use can be highlighted about the aging effects (Figure 11), which can be monitored thanks to periodic measurements, allowing the performance decay of the material to be highlighted due to chemical degradation linked to oxidative processes accelerated by exposure to ultraviolet light and ozone [34,35]. This is interesting from several points of view, for example, the decrease in progressive friction in the tire tread compounds, which affects road safety.

### 4.3. Optimizing Polymers’ Curing Cycles

Rubber is well known for being able to modify its viscoelastic properties when subjected to heating/cooling thermal cycles, also after the end of the vulcanization/production phase [36]. Knowing and controlling such effects can be useful in developing proper “curing cycles” with the aim of enhancing specific structural mechanisms. One of the application fields in which this is usually performed is again motorsport. Racing teams used to define curing strategies, applying heat a certain number of times through blankets, thermal guns, or specific ovens, although they usually need to work based on previous experiences or traditional routines.

**Figure 12:**
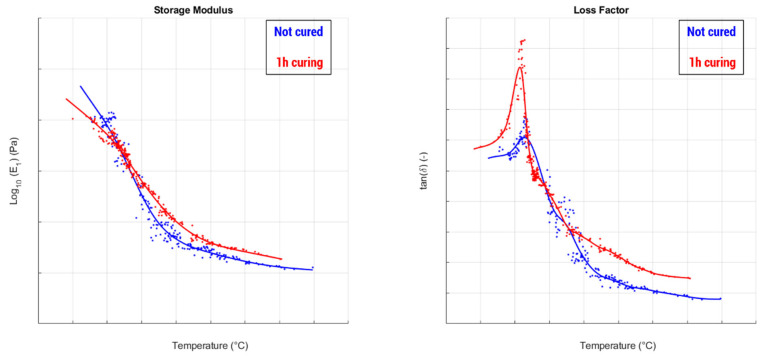

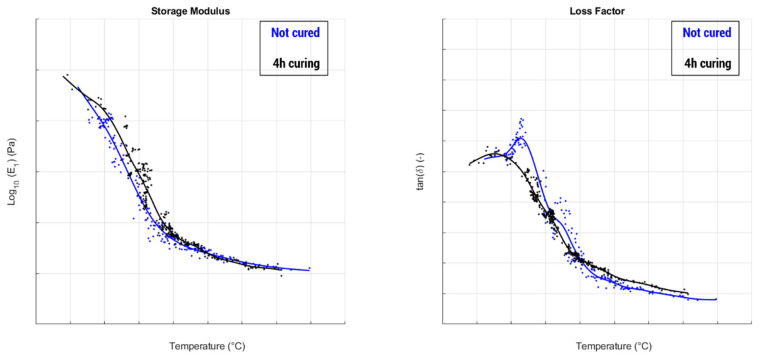
Results obtained after different thermal curing cycles (1 h in the top plots, 4 h in the bottom) for a racing tire, whose working zone is located in the high-temperature range of the viscoelastic curves (the diagrams are nondimensional due to confidentiality agreements).

The possibility to have an objectivation available of the viscoelastic effects caused by thermal cycles, provided by a nondestructive method that permits testing the same object multiple times, cycle after cycle, allows experimental sessions to be performed following a well-structured test plan involving various temperature levels, the duration of each cycle and number of cycles. As reported in the example described in Figure 12, one of the targets can be to find the optimal cycle duration that generates an increase in tan δ, considered responsible for the hysteretic frictional phenomena, keeping the E′, whose increase (hardening) tends to reduce the adhesive attitude of the material, at a stable level (in the example, it has been obtained using a 4 h curing cycle, while a 1 h cycle caused an undesired increase in E′, before it “relaxed” with a longer curing time).

The described procedures have also been adopted to carry out experimental tests aimed at defining the optimal vulcanization times by measuring rubber products after various curing cycles applied during the manufacturing process.

### 4.4. Assessing Tires Uniformity and Quality

Although one of the first targets of VESevo technology was the possibility of providing objective viscoelastic data on tires to those who could not get it, it was progressively discovered and used by the ones producing the tires themselves. Tire manufacturers were particularly interested in the capability to evaluate, in a fast and nondestructive test, the uniformity of the viscoelastic properties within a single tire, in various parts of its tread, and among different tires belonging to the same production batch.

In fact, although tire producers are becoming increasingly capable of guaranteeing a more repeatable production, it is well known that tires’ local nonuniformity (mainly due to the intrinsic complexity of the manufacturing and vulcanization process) affects ride, comfort, and safety [37] and that quality management tasks [22,38] represent a key factor in the competition towards safer and more sustainable vehicles, further emphasized by the labeling regulations released last year.

**Figure 13:**
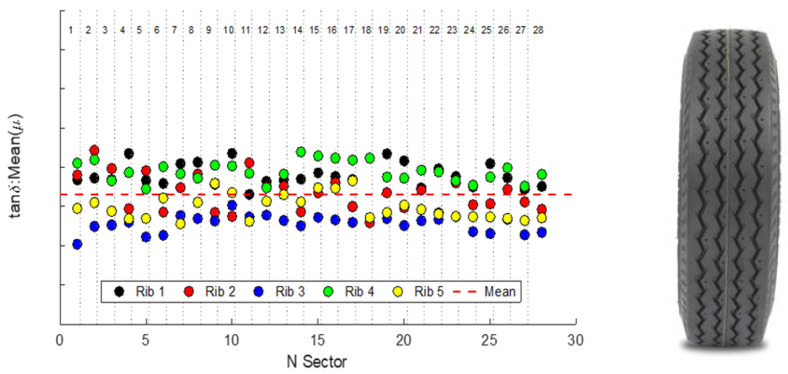
Results obtained using VESevo to test a single tire, characterized by a tread pattern showing five “ribs” in the lateral direction (as in the generic tire shown on the right) and divided into 28 sectors in the circumferential direction, exhibiting nonuniformities among the various ribs in terms of tan δ.

For the cited reasons, VESevo is progressively evolving as a real-time on-site technology that fits the requirements of the quality control industrial functions. One of the many applications in such a field is described in Figure 13, in which the result of a study on the uniformity of the viscoelastic properties among the various “ribs” of a five-rib tire tread is highlighted (the vertical axis is not shown due to confidentiality agreements with the tire manufacturer).

### 4.5. Not Only Tires: Assessing Viscoelastic Goods Uniformity and Quality

**Figure 14:**
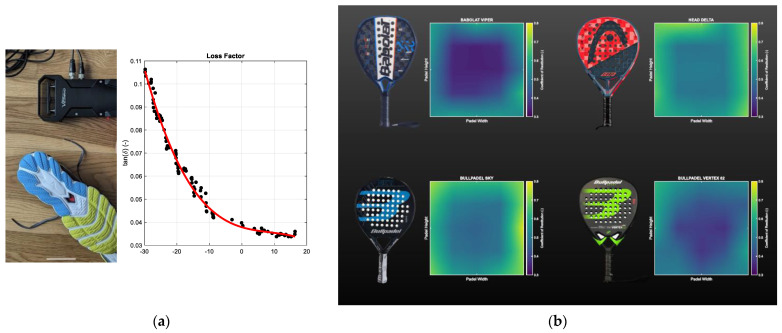

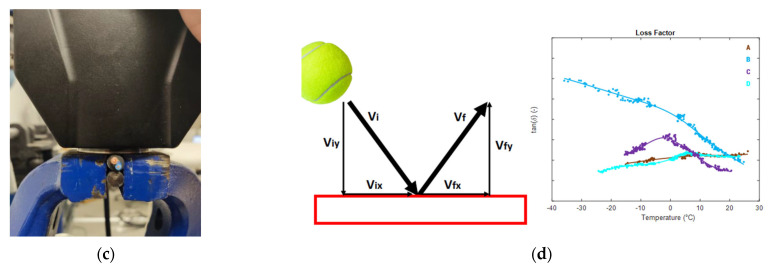
An overview of recent nonautomotive activities carried out involving VESevo technology: (**a**) testing soles of running shoes with its tan δ master curve; (**b**) testing padel rackets responses; (**c**) testing rubber cables; (**d**) testing tennis resin playing surfaces.

The scenario of using a related quality control assessment can be easily transferred to any industrial sector concerning the production of rubber/plastic goods characterized by viscoelastic behavior. Consequently, recent activities involving VESevo technology were carried out:

- In producing sports equipment (running shoes’ soles [39] (Figure 14a, padel rackets [40] (Figure 14b), tennis resin playing surfaces [41] (Figure 14d));
- In the rubber goods manufacturing industry (rubber seals and rings, rubber cables for electrical windings, rubber belts and cables for transmission (Figure 14c), rubber coating sheets, etc.);
- In the fashion market (artificial textiles and natural leather both exhibit viscoelastic behavior).

## 5. Conclusions

This paper introduces the VESevo (Viscoelasticity Evaluation System EVO), a nondestructive methodology based on an innovative device developed to characterize the viscoelastic properties of tire compounds, particularly focusing on tire treads. The VESevo overcomes the usual limitations due to DMA by providing a fast and portable system that measures the viscoelastic properties of tire compounds, allowing for comprehensive analyses throughout the tire’s lifecycle.

The device has been successfully applied in various scenarios, including motorsport, where it objectively characterizes tire compounds, validates tire uniformity, and correlates subjective driver feedback with objective data. Additionally, the VESevo technology enables the evaluation of the degradation of polymers over the entire lifecycle of a tire, providing insights into the progressive changes in the storage modulus and loss factor. This capability is crucial for monitoring the effects of wear, mechanical degradation, and aging on tire performance and safety.

Furthermore, the nondestructive testing procedure offered by VESevo proves valuable in optimizing polymers’ curing cycles, particularly in the context of motorsport teams seeking to enhance specific structural mechanisms. The ability to conduct repeated tests on the same object, cycle after cycle, allows for a structured experimental approach to defining optimal curing conditions.

The implications of this study extend beyond the tire and transportation industry, finding diverse applications and undergoing further investigations in various fields. The nondestructive and comprehensive nature of this methodology opens new avenues for research and development, fostering innovation in the field of viscoelastic material characterization and its applications in real-world scenarios.

## Data Availability

Data are contained within the article.
